# Supplementary material for: Expression of the novel maternal centrosome assembly factor Wdr8 is required for vertebrate embryonic mitoses
Source: Nat Commun. 2017 Jan 18;8:14090. doi: 10.1038/ncomms14090 (PMC5253655; doi:10.1038/ncomms14090)
Supplement: Supplementary Information — Supplementary Figures, Supplementary Tables, and Supplementary References [file ncomms14090-s1.pdf]

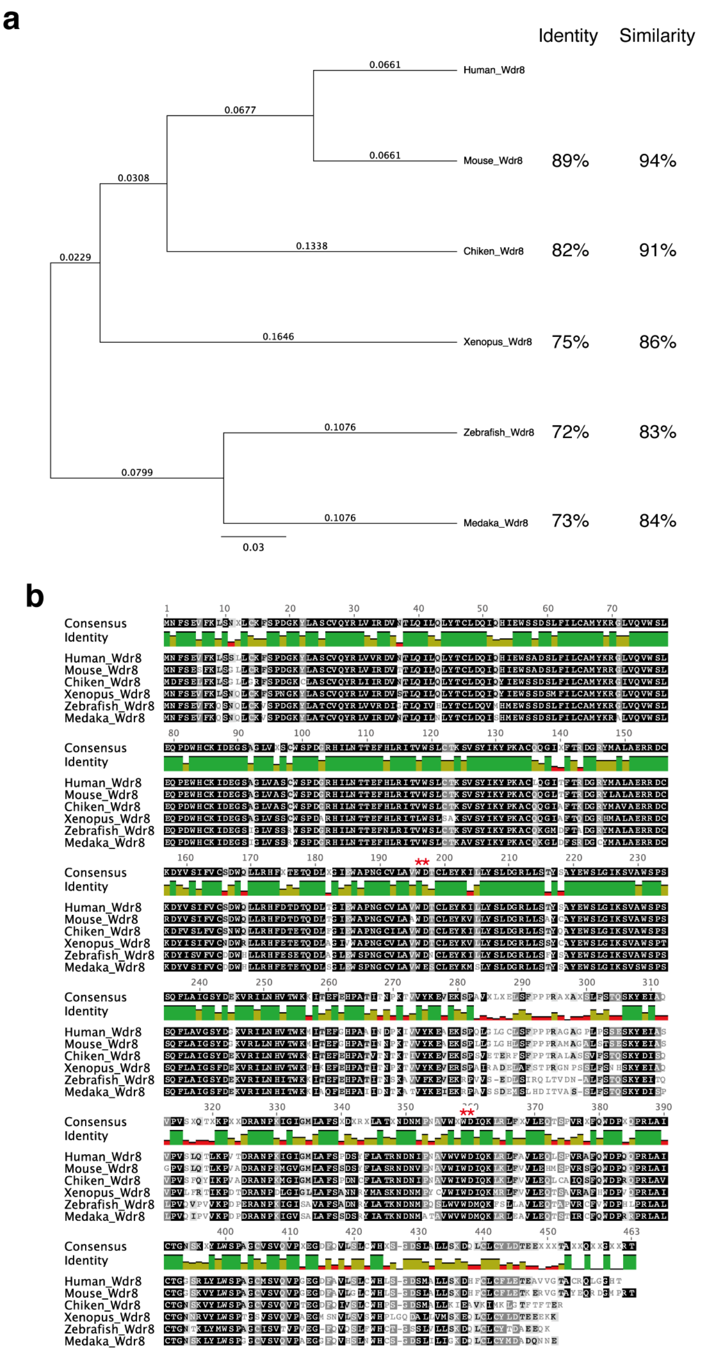

Supplementary Fig. 1 | Phylogenetic relationships of Wdr8 proteins among vertebrate species. a, Phylogenetic tree of Wdr8 proteins among vertebrate species. The percentages of identity and similarity of Wdr8 in each species relative to human Wdr8 are shown. Scale bar, 0.03/amino acid. **b,** Multiple alignment of Wdr8 proteins among vertebrate species. Red asterisks denote mutated amino acids in WD mutant variants used in this study (196/197AA and 359/360AA).

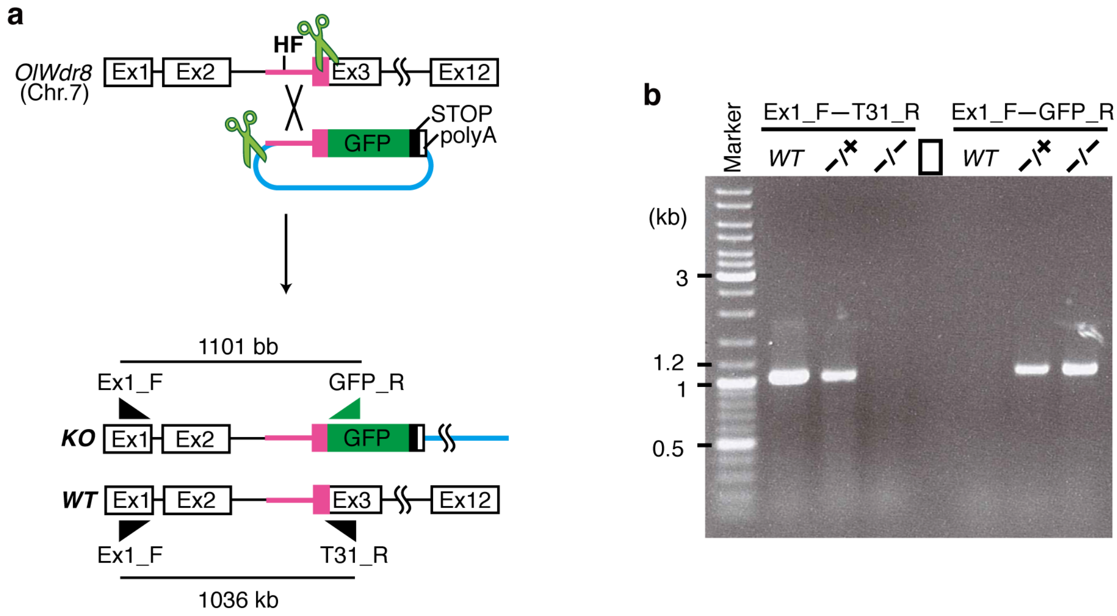

Supplementary Fig. 2 | CRISPR-Cas9 mediated knockout strategy to generate a *Wdr8*$^{-/-}$ medaka line. a, A sgRNA was designed to knockout (KO) exon-3 of *OlWdr8* by inserting a 5' homology flank (HF) and the *GFP* cDNA with a stop codon (see details in Materials and Methods). M, DNA marker. **b,** The screening of *Wdr8*$^{-/-}$ fish by genotype PCR. Two sets of primers, Ex1_F-T31_R and Ex1_F-GFP_R, were used to detect the wild-type (*WT*) and the KO genomic locus, respectively. Note that the *WT* locus was detected from genomic DNA of *WT* and *Wdr8*$^{-/+}$, but not from that of *Wdr8*$^{-/-}$. On the other hand, the KO locus was detected from genomic DNA of *Wdr8*$^{-/+}$ and *Wdr8*$^{-/-}$, but not from that of *WT*, due to the presence of the GFP insertion.

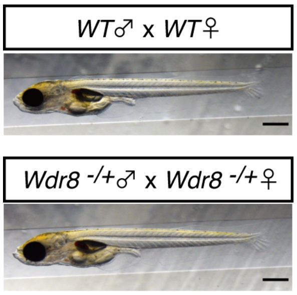

Supplementary Fig. 3 | External phenotype of Cab wild-type and *Wdr8*$^{-/-}$ hatchlings (F3 generation, see Materials and Methods). *Wdr8*$^{-/-}$ hatchlings rescued by maternal Wdr8 developed normally with no obvious external abnormalities, comparable to wild-type. Scale bars, 500 µm.

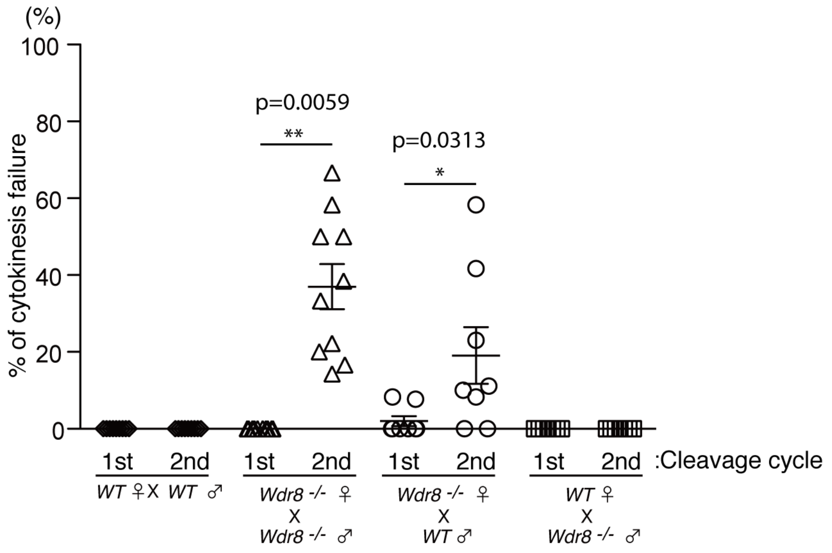

Supplementary Fig. 4 | Cytokinesis failure significantly increases from the second cleavage cycle on in zygotes from *Wdr8*$^{-/-}$ mothers. In comparison to the first cleavage cycle, cytokinesis failure increased significantly in the second cleavage, which led to three blastomeres instead of four blastomeres. Data represent mean ± s.e.m. The significances were analysed by Wilcoxon matched-paired signed rank test (two-tailed) and scored as follows: **; $P \leq 0.01$, *; $P \leq 0.05$. Please note that these data were simultaneously retrieved with the samples in Fig. 1b, and thus total number of zygotes and biological replicates are denoted in Supplementary Table 1. Each data point represents the percentage of cytokinesis failure counted from a clutch of individual different females. 9 clutches, *WT* ♀/ *WT* ♂; 10 clutches, *Wdr8*$^{-/-}$ ♀/ *Wdr8*$^{-/-}$ ♂; 8 clutches, *Wdr8*$^{-/-}$ ♀/ *WT* ♂; 7 clutches, *WT* ♀/ *Wdr8*$^{-/-}$ ♂.

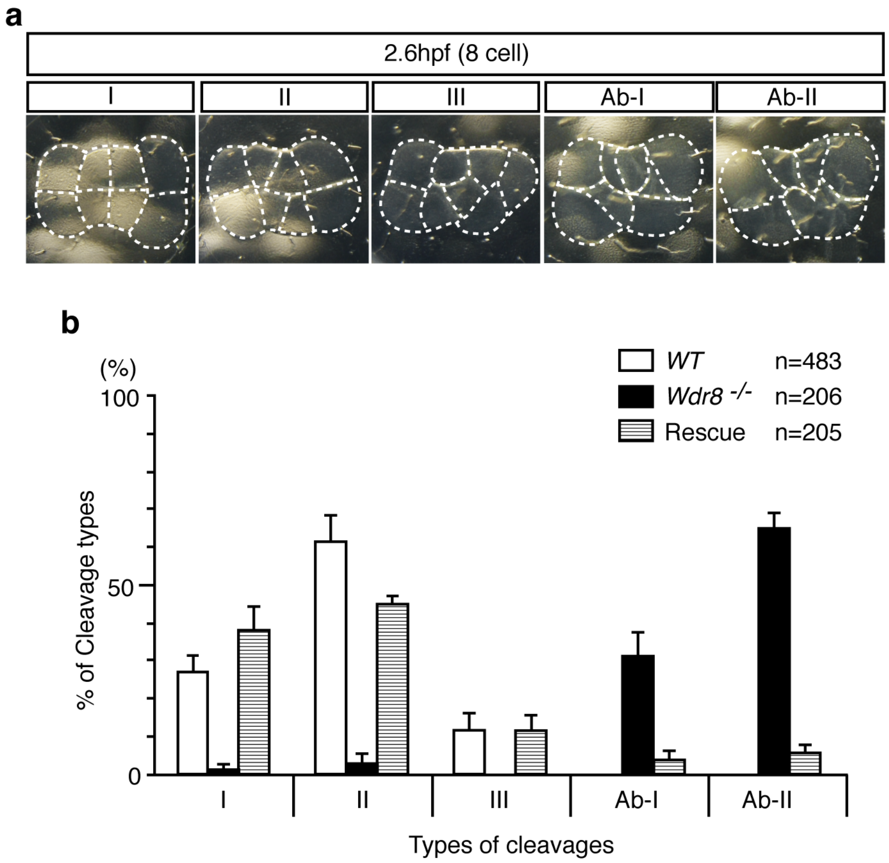

Supplementary Fig. 5 | Rescue of abnormal cleavage divisions of *Wdr8*$^{-/-}$ zygotes by EYFP-huWdr8 expression. a, Classification of cleavage types at 2.6hpf (8-cell stage in *WT*). 8-cell *WT* zygotes showed Type I-III cleavages with regard to the degree of axial symmetry (I; highly symmetric, II; partially symmetric, III; asymmetric)[1]. Abnormal cleavages were classified as Ab-I, i.e. 8 cells with various sizes of blastomeres, or Ab-II, i.e. 5-7 cells instead of 8, possibly caused by cytokinesis failure or multipolar spindles, which cannot be classified as types I-III. Dotted lines represent developing zygotes onto the yolk. **b**, The expression of EYFP-huWdr8 efficiently rescued abnormal cleavages of *Wdr8*$^{-/-}$ to the three types of cleavages as in *WT*. Data represent mean ± s.d. n, total number of zygotes from four (*Wdr8*$^{-/-}$, Rescue) or five (*WT*) independent experiments.

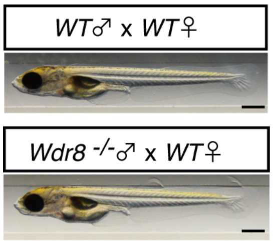

Supplementary Fig. 6 | External phenotype of the hatchlings from *Wdr8*$^{-/-}$ fathers in comparison to wild-type hatchlings. As in Supplementary Fig. 3, no obvious differences could be detected, demonstrating that paternal Wdr8 is not implicated in both early and later stage development of medaka. Scale bars, 500 µm.

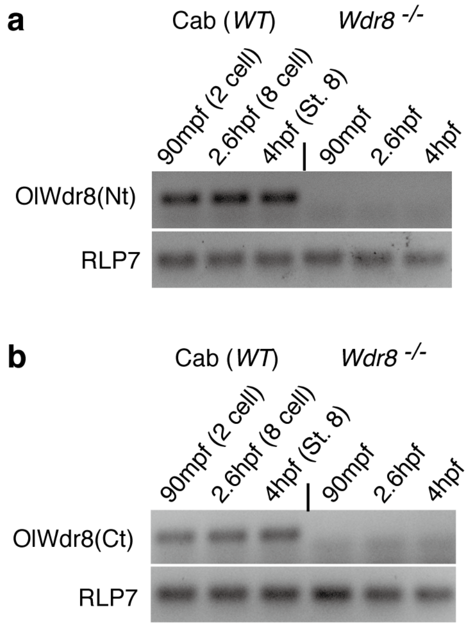

Supplementary Fig. 7 | OlWdr8 expression in *WT* and *Wdr8*$^{-/-}$ zygotes' development analysed by RT-PCR using specific probes against regions of the Wdr8 transcript encoding stretches in the N-terminal (Nt) or the C-terminal (Ct) part of the protein. 90mpf, 2.6hpf, and 4hpf correspond to 2-cell, 8-cell, and St.8 (64-128 cells) in Cab *WT*, respectively. Ribosomal protein L7 (RLP7) expression is shown as a reference control[2]. Note that neither the N-terminal (**a**, Nt) nor the C-terminal (**b**, Ct) encoding stretches of the OlWdr8 transcript could be amplified in *Wdr8*$^{-/-}$ zygotes, while OlWdr8 was maternally expressed from 90mpf to 4hpf at almost the same level in WT zygotes. This demonstrates that full-length Wdr8 mRNA is absent in *Wdr8*$^{-/-}$ zygotes.

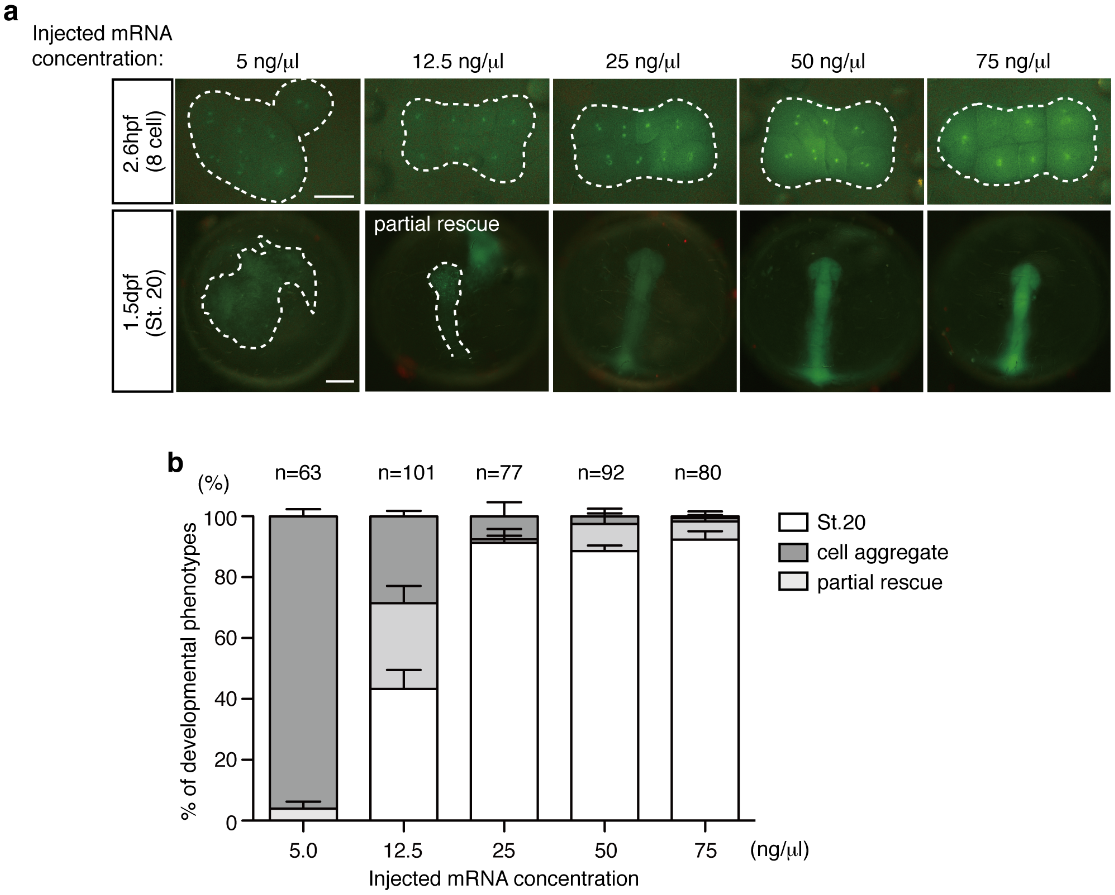

Supplementary Fig. 8 | Dose dependent rescue of *Wdr8*$^{-/-}$ zygotes by injection of increasing amounts of EYFP-huWdr8 mRNA. a, Representative external phenotypes of *Wdr8*$^{-/-}$ zygotes injected with EYFP-huWdr8 mRNA at concentrations ranging from 5 to 75 ng/μl. At all concentrations, centrosomal localisation of EYFP-huWdr8 could be seen suggesting that centrosomal accumulation of EYFP-huWdr8 is not saturated (2hpf). Although EYFP-huWdr8 localised to the centrosome (**a**, 2.6hpf), the concentrations of 5 ng/μl and 12.5 ng/μl mRNA were insufficient to rescue *Wdr8*$^{-/-}$ zygotes (**a**, 1.5dpf and **b**). Dotted lines represent developing zygotes onto the yolk. **b**, The rescue efficiency of EYFP-huWdr8 for *Wdr8*$^{-/-}$ zygotes appeared to follow a non-linear dose response typical for a catalytic activity with an apparent inflection point at around 12.5 ng/μl. Scale bars, 200 μm. The data represent mean ± s.d. n, total number of zygotes from four independent experiments.

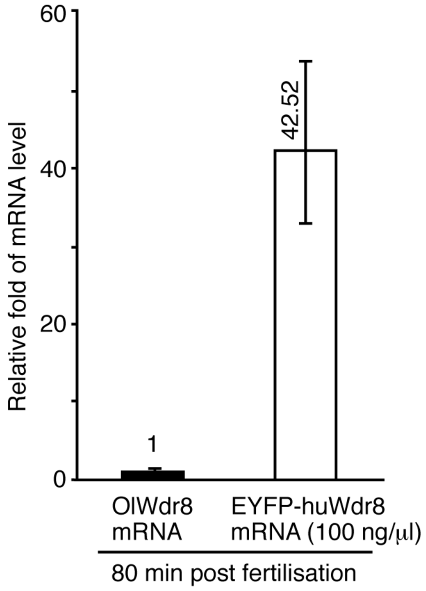

Relative fold of mRNA level		range
Cab (*WT*)	1	1 + 0.06<br>1 - 0.06
Wdr8 $^{-/-}$	NA	NA
100 ng/ul EYFP-huWdr8 / *Wdr8* $^{-/-}$	42.52	42.52 + 11.48<br>42.52 - 9.42

Supplementary Fig. 9 | Ratios of EYFP-huWdr8 and OlWdr8 mRNAs in the rescued zygotes. Quantification of EYFP-huWdr8 mRNA relative to endogenous OlWdr8 mRNA in the rescued *Wdr8*$^{-/-}$ zygotes was performed by real-time PCR. EYFP-huWdr8 corresponding to about 40-fold endogenous OlWdr8 mRNA was introduced into *Wdr8*$^{-/-}$ zygotes at 80mpf. Note that 80mpf corresponds to the end of the first or the entry into the second mitosis, when the centrosome/spindle abnormalities begin in *Wdr8*$^{-/-}$ zygotes (See Fig. 1b and 60-70mpf in Supplementary Fig. 10). Therefore, 80mpf is the critical point to rescue *Wdr8*$^{-/-}$ zygotes. The data represent mean ± s.d. from four independent RT-PCR experiments, NA, not available due to absent amplicon detection by real-time PCR.

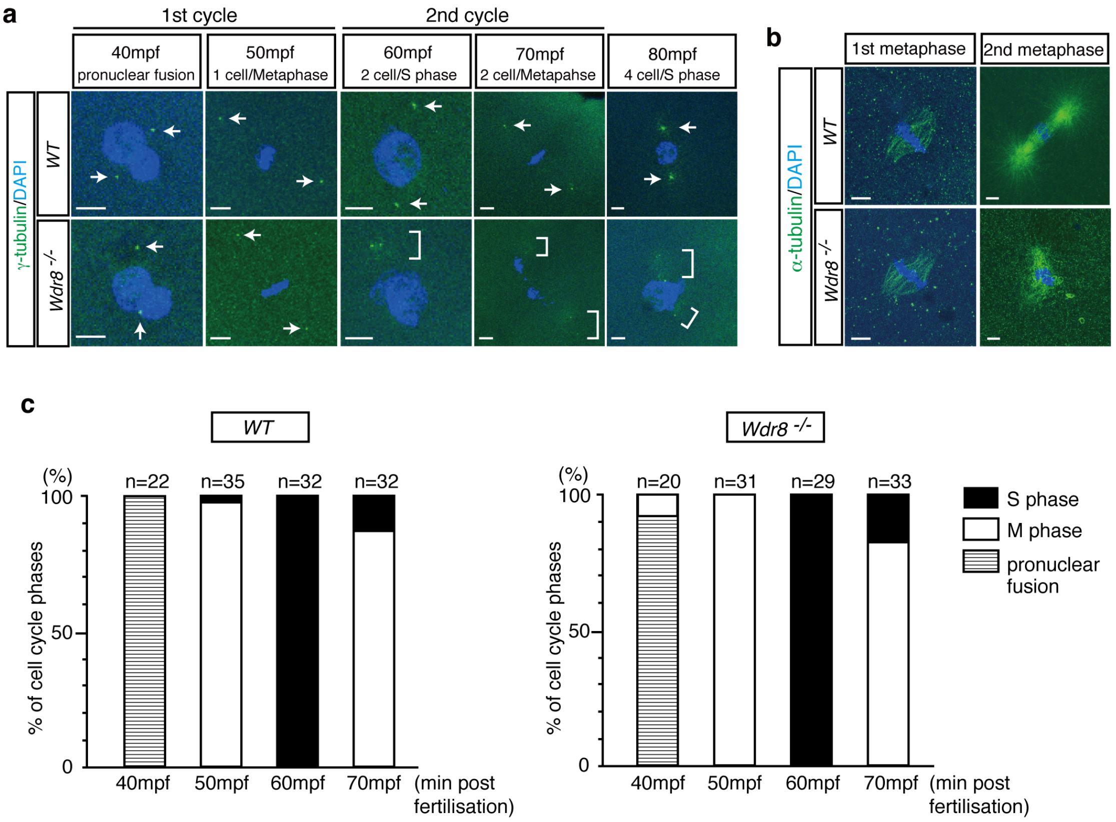

Supplementary Fig. 10 | Maternal Wdr8 is essential from the second mitosis on but not required in first mitosis due to a possible paternal contribution. a, The centrosome and chromosome behaviors in the first and second embryonic cell cycle of *WT* and *Wdr8*$^{-/-}$ zygotes. In the first cell cycle, both *WT* and *Wdr8*$^{-/-}$ showed normal pronuclear fusion and metaphase figures with two separated centrosomes (arrows). In contrast, from the second cell cycle on, *Wdr8*$^{-/-}$, but not *WT*, exhibited incomplete separation (60’, square bracket) or dispersion of the centrosomes (70’ and 80’, square brackets), causing chromosome alignment defects at metaphase (70’). **b**, The mitotic bipolar spindle in the first and second embryonic cell cycle of WT and *Wdr8*$^{-/-}$ zygotes. The first mitotic bipolar spindle in *Wdr8*$^{-/-}$ was comparable to that of *WT*. In contrast, as seen for centrosome structure (**a**), abnormal spindle assembly emerged from the second cell cycle. **c**, Timing of first and second embryonic cell cycles in *WT* and *Wdr8*$^{-/-}$. DAPI/pH3 stained chromosomes were analysed to determine cell cycle phases. In both WT and *Wdr8*$^{-/-}$, oscillation of the cell cycle by alternating between S and M phases was almost synchronous, indicating that there was no apparent delay in the first and the second mitosis. Scale bars, 10 μm (**a**, **b**), n, total number of immunostained zygotes analysed (**a**, **b**, **c**).

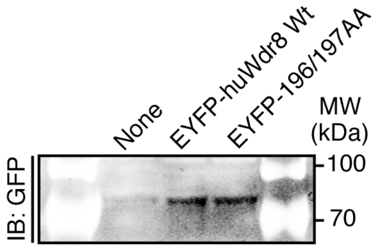

Supplementary Fig. 11 | Western blot analysis to compare the protein levels between EYFP-huWdr8 and EYFP-196/197AA. Expression levels of both proteins were almost equivalent, demonstrating that the mutations in WD40 domain did not affect protein stability.

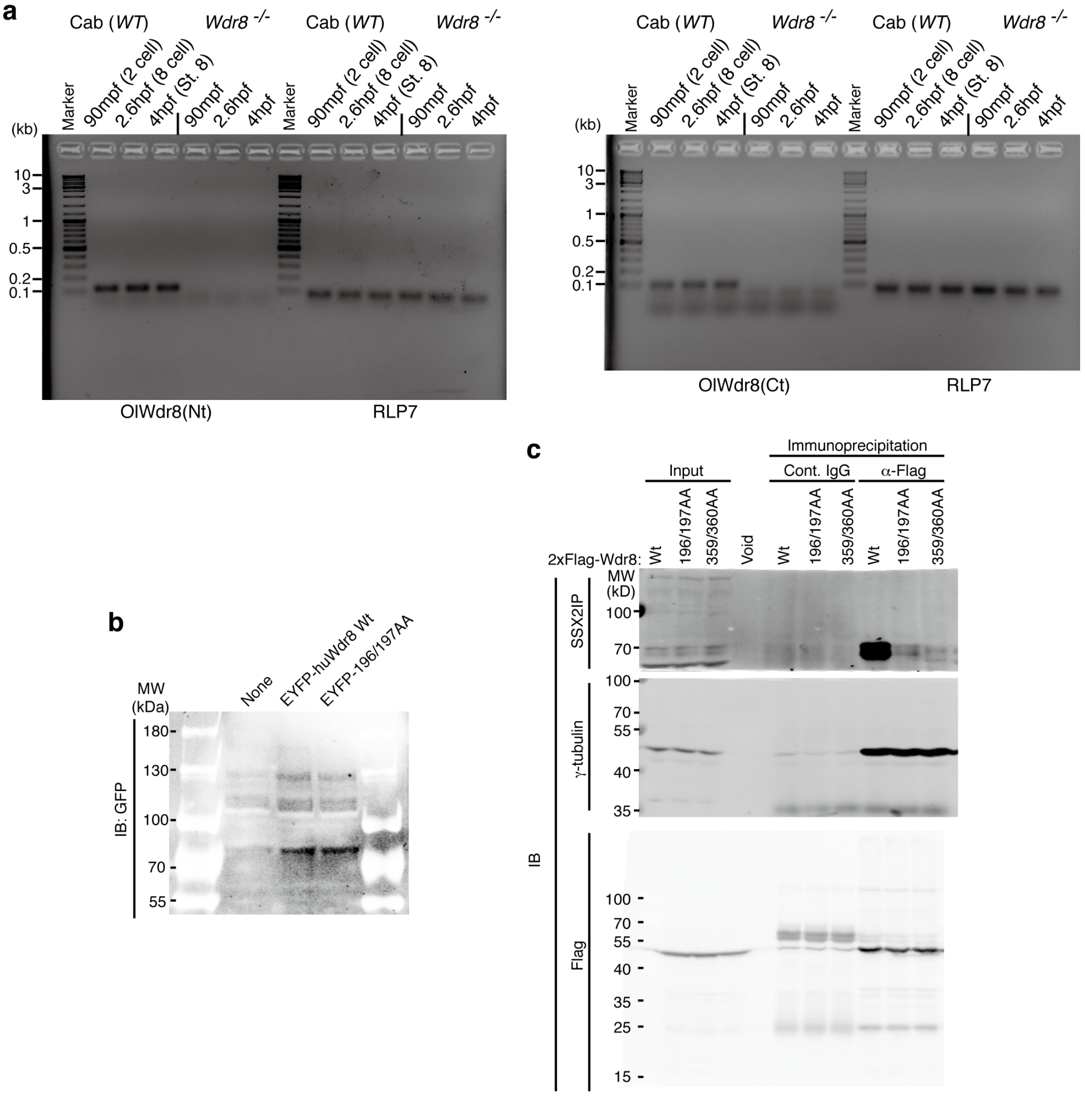

Supplementary Fig. 12 | Scans of original gels and Western blots. a, Uncropped gels in Supplementary Fig. 7. **b**, **c**, Uncropped blots in Supplementary Fig. 11 (**a**) and Fig. 5c (**b**).

The average cleavage timings and their variances shown in Fig. 1b

	Cleavage divisions (min post fertilisation ± S.D.)				Total zygotes	Biological replicates
Mating combination	1st	2nd	3rd	4th		
WT ♀/ *WT* ♂	88 ± 2.1	123.7 ± 5.8	153.9 ± 6.3	182.4 ± 6.1	n=112	9
WT ♀/ *Wdr8*$^{-/-}$ ♂	87.6 ± 3.2	113.8 ± 4.9	152.9 ± 3.8	183.5 ± 14.2	n=72	7
Wdr8$^{-/-}$ ♀/ *Wdr8*$^{-/-}$ ♂	89.3 ± 6.2	126 ± 14.2	167.6 ± 16.3	196.8 ± 14.2	n=82	10
Wdr8$^{-/-}$ ♀/ *WT* ♂	90.9 ± 5.3	127.1 ± 10.2	167.2 ± 8.4	206.6 ± 9.9	n=87	8

Supplementary Table 1 | The average cleavage timings and their variances in offspring generated under the individual mating combinations shown in Fig. 1b. The data represent mean (mpf) ± s.d. Total numbers of zygotes and biological replicates are denoted in the table.

Wdr8-sgRNA target site and the potential off-target sites in the entire medaka genome

Chr.	start (bp)	end (bp)	strand	MM	target sequence	PAM	alignment	distance*	Gene name	gene ID
7	8,181,467	8,181,489	+	0	GGTCTCTCGA GCAGCCGGAC	TGG	IIIIIIII[IIIIIIII IIII]PAM	0	Exonic wrap73	ENSORLG00000004450
23	965,852	965,874	+	4	GGTCACATGT GCAGCCGGAC	AGG	IIII-I--[I-IIIIIIIIII]PAM	482	Intronic MAPK11	XLOC_015134
7	22,850,530	22,850,552	+	4	CGTCCCTCGA GCATCCGCAC	GGG	-III-III[IIIII-III-II]PAM	0	Exonic csrnp2	ENSORLG00000015651

Supplementary Table 2 | The chosen Wdr8-sgRNA target site has only two potential off-target sites in the entire medaka genome. Search-parameters (see [44]) for further details): core length (in parenthesis) = 12, max. core mismatches = 2, max. total mismatches (MM) = 4, PAM = NGG. *, distance to next exon.

Supplementary References

1. Kraeussling, M., Wagner, T. U. & Schartl, M. Highly asynchronous and asymmetric cleavage divisions accompany early transcriptional activity in pre-blastula medaka embryos. *PLoS One* **6**, e21741 (2011).
2. Zhang, Z. & Hu, J. Development and validation of endogenous reference genes for expression profiling of medaka (Oryzias latipes) exposed to endocrine disrupting chemicals by quantitative real-time RT-PCR. *Toxicol. Sci.* **95**, 356-368 (2007).
